# Circulating Endothelial Progenitor Cells Present an Inflammatory Phenotype and Function in Patients With Alcoholic Liver Cirrhosis

**DOI:** 10.3389/fphys.2018.00556

**Published:** 2018-05-22

**Authors:** Savneet Kaur, Rashi Sehgal, Saggere M. Shastry, Geoffrey McCaughan, Helen M. McGuire, Barbara Fazekas St de Groth, Shiv Sarin, Nirupma Trehanpati, Devanshi Seth

**Affiliations:** ^1^Department of Molecular and Cellular Medicine, Institute of Liver and Biliary Sciences, New Delhi, India; ^2^Liver Injury and Cancer, Centenary Institute of Cancer Medicine and Cell Biology, Camperdown, NSW, Australia; ^3^Sydney Medical School, University of Sydney, Sydney, NSW, Australia; ^4^Ramaciotti Facility for Human Systems Biology, University of Sydney, Sydney, NSW, Australia; ^5^Discipline of Pathology, University of Sydney, Sydney, NSW, Australia; ^6^Drug Health Services, Royal Prince Alfred Hospital, Camperdown, NSW, Australia; ^7^Faculty of Medicine, The University of Sydney, Sydney, NSW, Australia

**Keywords:** angiogenesis, alcoholic liver cirrhosis, endothelial progenitor cells, inflammation, mass cytometry CyTOF, CD45, CD133, CD34

## Abstract

**Background and Aim:** Endothelial progenitor cells (EPCs) have been implicated in liver injury and repair. However, the phenotype and potential of these heterogenous EPCs remain elusive. In particular, their involvement in the pathogenesis of alcoholic liver cirrhosis (ALC) remains unclear. The current study extensively characterized the phenotype and functions of EPCs to understand their role in ALC pathogenesis.

**Methods:** Circulating EPCs were identified as CD34+CD133+CD31+ cells by flow cytometer in ALC patients (*n* = 7) and healthy controls (HC, *n* = 7). A comprehensive characterization of circulating EPCs using more than 30 phenotype markers was performed by mass cytometer time of flight (CyTOF) in an independent cohort of age and gender matched ALC patients (*n* = 4) and controls (*n* = 5). *Ex vivo* cultures of circulating EPCs from ALC patients (*n* = 20) and controls (*n* = 18) were also tested for their functions, including colony formation, LDL uptake, lectin binding and cytokine secretion (ELISA).

**Results:** Three distinct populations of circulating EPCs (CD34+CD133+CD31+) were identified, classified on their CD45 expression (negative: CD45^−^; intermediate: CD45^int^; high: CD45^hi^). CD45^int^ and CD45^hi^ EPCs significantly increased in ALC patients compared to controls (*p*-val = 0.006). CyTOF data showed that CD45^hi^ EPCs were distinct from CD45^−^ and CD45^int^ EPCs, with higher expression of T cell and myeloid markers, including CD3, CD4, HLA-DR, and chemokine receptors, CCR2, CCR5, CCR7, and CX3CR1. Similar to circulating EPCs, percentage of CD45^hi^CD34+CD31+ EPCs in *ex-vivo* cultures from patients, were significantly higher compared to controls (*p* < 0.05). Cultured EPCs from patients also showed increased LDL uptake, lectin binding and release of TNF-alpha, RANTES, FGF-2, and VEGF.

**Conclusions:** We report the first extensive characterization of circulating human EPCs with distinct EPC subtypes. Increase in CD45^hi^ EPC subtype in ALC patients with enhanced functions, inflammatory cytokines and angiogenic mediators in patients suggests an inflammatory role for these cells in ALC.

## Introduction

Alcoholic liver disease (ALD) remains the leading cause of chronic liver diseases and mortality worldwide (WHO, 2014). Excessive alcohol consumption induces steatosis and prolonged ingestion may lead to steatohepatitis, fibrosis, cirrhosis, and hepatocellular carcinoma (Toshikuni et al., 2014). However, the underlying mechanisms contributing to the pathogenesis of ALD progressing to alcoholic liver cirrhosis (ALC) are only partially understood (Seth et al., 2010).

Given the extensive changes in the vascular architecture of liver during injury and repair, angiogenesis and increased blood vessel growth are suspected to significantly contribute to inflammation, fibrogenesis, and disease progression in chronic liver diseases (Amarapurkar et al., 2007; Bocca et al., 2015). Long-term ethanol (1.6 g/kg body wt/day) consumption for 36 weeks has been associated with severe oxidative stress and immunological alterations that trigger angiogenesis through coordinated action of a variety of mediators (Das et al., 2012). Ethanol is also known to increase HIF-1alpha and VEGF levels in both murine and human endothelial cells, resulting in enhanced intracellular signal transduction, expression of angiogenic markers, tube formation and wound healing, which are the hallmarks of the angiogenesis process (Raskopf et al., 2014). In normal liver with intact parenchyma and vasculature, the neovessel formation or angiogenic signals are mediated by the liver sinusoidal endothelial cells (LSECs). However, in conditions of liver injury and regeneration the angiogenic signals may also be mediated by other non-LSEC sources such as hematopoetic cells (HSCs) and bone marrow (BM)-derived endothelial progenitor cells (BM-EPCs) (DeLeve, 2013).

These cells migrate to the injured tissue via angiogenic signals and then contribute to endothelial repair and neo-vascularization by distinct mechanisms (Asahara et al., 2011). The precise role of EPCs in liver angiogenesis and fibrosis has just begun to be understood. In our previous study, we reported that circulating EPCs are significantly increased in patients with cirrhosis and that cultured EPCs interacted with the resident LSECs resulting in an enhanced angiogenesis/neovessel formation via secretion of angiogenic factors, VEGF and PDGF-BB *in vitro* (Kaur et al., 2012). Recently, we reported that BM-EPCs migrate and home to the liver during CCL4-induced liver injury *in vivo* in mice (Garg et al., 2017).

However, EPCs represent a heterogenous population of cells and the lineage and precise phenotype of these cells remain largely elusive. Given an important contribution of circulating EPCs in liver angiogenesis, the current study comprehensively characterized circulating and cultured EPCs in patients with ALC to understand their potential role in this disease.

## Materials and methods

### Study subjects and collection of blood samples

Alcoholic Liver Cirrhosis (ALC, *n* = 27) patients diagnosed on the basis of clinical and radiological presentation and age matched healthy controls without any liver disease (HC, *n* = 25) were recruited in this study (Table 1). Heparin-treated blood samples (*n* = 7 ALC, *n* = 7 HC for flow cytometry; *n* = 20 ALC, *n* = 18 HC for EPC culture experiments), were collected between 2015 and 2017 in the out-patient department (OPD) of the Institute of Liver and Biliary Sciences (ILBS), New Delhi. Peripheral Blood Mononuclear Cells (PBMCs) were isolated from blood using Ficoll-Hypaque (Sigma-Aldrich, USA) by density gradient centrifugation. The study was approved by the institutional ethics committee of ILBS (IEC/IRB no. 35/5) and informed consent was obtained from all the subjects. Another independent cohort from Sydney, Australia, was also recruited to perform extensive immunophenotyping by mass cytometry described below. PBMCs were isolated from heparin-treated whole blood using Ficoll Paque Plus (GE Healthcare, Uppsala, Sweden) and stored in vapor phase liquid nitrogen till further analysis. Informed consent was obtained from all patients (*n* = 4) and healthy controls (*n* = 5) under protocol HREC/11/RPAH/88 and HREC/15/RPAH/380 approved by Sydney Local Health District Human Research Ethics Committee.

**Table 1:** Clinico-pathological features of patients.

	**Healthy controls (HC)**	**Alcoholic cirrhosis (ALC)**	
*N*	25	27	
	**Median (Range)**		***P-*****value**
Age (years)	38 (31–41)	43 (20–61)	0.06
Male: Female	28:3	53:2	–
ALT/SGPT (IU/L)	16 (12–19)	36 (14–350)	0.0000^*^
AST/SGOT (IU/L)	9 (6–15)	76.4 (16–1,460)	0.0001^*^
SAP (IU/L)	36 (33–40)	99.50 (12–332)	0.0000^*^
GGT (IU/L)	11 (8–16)	38 (11–449)	0.0000^*^
Total Protein (g/dL)	7 (6.2–7)	7 (3–8.4)	0.10
Serum Albumin (g/dL)	4 (3.6–4.1)	2.5 (1.4–3.5)	0.06
Serum Globulin (g/dL)	2 (2.1–2.8)	4 (1.3–5.4)	0.06
PT (s)	12 (12–12.4)	19 (10–36.2)	0.07
INR	1 (1–1.2)	2 (0.93–4.77)	0.056
Bilirubin (mg/dL)	1 (0.4–0.9)	4 (1.5–35.1)	0.058
AFP (ng/ml)	0	6.3	0.001^*^
Hb (g/dL)	–	20 (10–27)	0.20
TLC (×10^9^ per liter)	15 (13–16)	10 (7–12.5)	–
Lymphocytes (%)	–	8 (2.8–22.3)%	0.057
Monocytes (%)	30 (22–36)	16 (5–68)	0.08
Neutrophils (%)	6 (3–9)	8 (2–23)%	0.06
Eosinophils (%)	54 (45–70)	72 (6–92)	0.60
Platelet Count (×10^9^ /liter)	3 (2–5)	3 (1–18)	–

*AFP, Alfa fetoprotein; ALT, Alanine aminotransferase; AST, Aspartate aminotransferase; GGT, Gamma-glutamyl transferase; HB, hemoglobin; INR, International Normalized Ratio; IU/L, International unit/liter; PT, Prothrombin time; SAP, Serum alkaline phosphatise; TLC, Total leukocyte count*.

### Phenotypic characterization of endothelial progenitor cells (EPCs) by flow cytometry

Endogenous circulating EPCs and cultured EPCs were identified and characterized by flow cytometry using progenitor, hematopoietic, and endothelial markers. For identification of circulating EPCs, antibodies against human CD45, CD34, CD133, and CD31 and for cultured EPCs, CD45, CD31, and Vegfr2 were used (Table 2). Gating for live EPCs was done in the region of lymphocytes and monocytes. Single color profiles for different flourochromes were used as controls (Supplementary Figure 1A). Multicolor flow cytometry was performed using FACS Verse (BD Biosciences, San Jose, CA, USA) and a minimum of 1 million events were acquired. Analysis of flow cytometry data was performed using Flow-Jo software (BD Biosciences, CA, USA).

**Table 2:** Flow cytometry antibody panel.

**Antibody**	**Antibody clone**	**Antibody source**	**Fluorochrome**
CD31	WM59	BD Biosciences	Alexa 647
CD34	563	BD Biosciences	PE
CD45	2D1	BD Biosciences	Percp
CD133	Ab16518	Abcam	Unconjugated used with IgG anti-rabbit FITC
Vegfr2	89106	BD Biosciences	PE

### Immunophenotypic characterization of endothelial progenitor cells (EPCs) by mass cytometry

PBMCs cryopreserved in vapor phase liquid nitrogen were thawed and washed with RPMI1640 (Thermo Fisher Scientific, Waltham, MA, USA) supplemented with 10% heat activated fetal calf serum, 2 mM L-Glutamine, 25 μM 2-Mercapto-ethanol, and 1,000 units/L of Penicillin/Strep (Invitrogen). Two million PBMCs were stained for mass cytometry (CyTOF) analyses as described (Bendall et al., 2011; Newell et al., 2012), with ~36 metal tagged antibodies listed in Table 3. Briefly, PBMCs were stained with 1.25 μM cisplatin in PBS for 3 min at room temperature and quenched with facs buffer (PBS with 5% fetal calf serum). Cells were incubated for 30 min at 4°C with a 50 μl cocktail of metal-conjugated antibodies targeting surface antigens. All antibodies (Table 3), with the exception of fluorescently labeled or metal tagged antibodies from Fluidigm, were conjugated with MaxPar X8 labeling reagent kits (Fluidigm) according to the manufacturer's instructions by the Ramaciotti Facility for Human Systems Biology, Sydney, Australia. Following wash with FACS buffer, cells were fixed in 4% paraformaldehyde containing DNA intercalator (0.125 μM Iridium-191/193; Fluidigm). After multiple washes with FACS buffer and MilliQ water, cells were filtered through a 35 μm nylon mesh and diluted to 800,000 cells/ml in MilliQ with EQ beads (Fluidigm) diluted 1 in 10. Cells were acquired at a rate of 200–400 cells/s using a CyTOF 2 Helios upgraded mass cytometer (Fluidigm, Toronto, Canada). Cells were normalized for signal intensity of EQ beads using the Helios software. FlowJo X 10.0.7r2 software (FlowJo, LLC, Ashland, OR, USA) was used to gate populations of interest and export median signal intensity for markers across populations and study participants which was then visualized by heatmap (Prism), ordered by average marker intensity in CD34+CD45hi population. Additionally, the t-stochastic neighborhood embedding (t-SNE) algorithm (implemented in FlowJo as a PlugIn) was utilized to perform dimensionality reduction and visualization of immune subpopulations across samples. A fixed number of 20,000 cells was sampled (without replacement) from total live events of a healthy donor and combined with all EPC events (CD34+ and CD31+, distinguished for CD45^−^, CD45^inter^, and CD45^hi^ expression) pooled across study participants for analysis. The resulting t-SNE plots were visualized by overlaying populations gated in FlowJo, and colored by the various subsets. All markers stained by antibodies were used for clustering.

**Table 3:** Mass cytometry antibody panel information (alphabetical order).

**Antibody**	**Antibody clone**	**Antibody source**	**Isotope/Fluorochrome**
Anti-APC	APC003	Fluidigm	Lu176
Anti-FITC	FIT-22	Fluidigm	Nd144
Anti-PE	PE001	Fluidigm	Nd145
CCR5	HEK/1/85a	Biolegend	FITC
CD117	104D2	Biolegend	Nd143
CD11b	ICRF44	Biolegend	Bi209
CD11c	Bu15	Biolegend	In115
CD127	A019D5	Biolegend	Ho165
CD133	Clone 7	Biolegend	APC
CD14	M5E2	BD Biosciences	Gd160
CD16	3G8	BD Biosciences	Nd148
CD183 (CXCR3)	G025H7	Biolegend	Dy163
CD184 (CXCR4)	12G5	BD Biosciences	Lu175
CD19	HIB19	Biolegend	Nd142
CD192 (CCR2)	K036C2	Biolegend	Eu151
CD197 (CCR7)	G043H7	Biolegend	Tb159
CD25	M-A251	Biolegend	Tm169
CD27	M-T271	BD Biosciences	Pr141
CD283 (TLR3)	TLR-104	Biolegend	Sm147
CD284 (TLR4)	HTA125	Biolegend	Sm149
CD3	UCHT1	BD Biosciences	Er170
CD31	WM59	Biolegend	Gd155
CD33	WM53	Fluidigm	Gd158
CD335 (NKp46)	9E2	Biolegend	Yb173
CD337 (NKp30)	P30 15	BD Biosciences	Nd150
CD34	581/CD34	BD Biosciences	Er166
CD38	HIT2	Biolegend	Er167
CD4	SK3	Biolegend	Er168
CD40	HB14	Miltenyi	Yb171
CD45	HI30	Fluidigm	Gd154
CD45RO	UCHL1	Biolegend	Dy161
CD57	HCD57	Biolegend	La139
CD66b	80H3	Fluidigm	Sm152
CD68	KP1	Bio-Rad	Eu153
CD69	FN50	Fluidigm	Er162
CD86	IT2.2	BD Biosciences	Gd156
CD8A	RPA-T8	Biolegend	Nd146
CX3CR1	2A9-1	Biolegend	Er164
HLA-DR	L243	Biolegend	Yb174
IgM	MHM-88	Biolegend	Yb172
Vegrf2	89106	BD Biosciences	PE
**LIVE/DEAD MARKER**			
Cisplatin		Fluidigm	Pt194/195

### Functional characterization of EPCs

#### Colony formation in *ex vivo* cultures of EPCs

PBMCs were isolated from peripheral blood (ALC, *n* = 20; HC, *n* = 18) using Ficoll-Hypaque. For EPC cultures, PBMCs were resuspended in endothelial basal medium (EBM)- 2 (Lonza, USA) and endothelial growth supplement (EGM-2) (Single Quots, Lonza, USA) supplemented with 10% FBS, and seeded in a 60 mm dish pre-coated with fibronectin (1μg/ml) at a final density of 5 × 10^6^ cells/ml. After 24 h, non-adherent cells were removed and fresh EBM-2 media was added for culturing of adherent cells_._ Media was changed daily for 7 days and every other day thereafter. After 10–15 days, cells were checked for characteristic EPC morphology and colony formation as defined (Hill et al., 2003). An average of 5–6 fields in 3–4 wells of each sample (ALC or HC) was taken to ensure accurate counting of the EPC colonies. Cultured EPCs were trypsinized and characterized (CD45, CD34, CD31, and Vegfr2) at day 10 by flow cytomtery as described above.

#### LDL uptake assay

The ability of cultured EPCs to take up acetylated low density lipoprotein (ac-LDL) and UEA-lectin was performed. Briefly, EPCs at seeding density of 2 × 10^4^ cells/well were plated onto fibronectin coated 24-well tissue culture plate. Cells were allowed to adhere for 8–24 h prior to assaying for ac-LDL and lectin uptake. Culture medium was removed, cells were washed with 500 μl 1xPBSand incubated for 4 h in Dil ac-LDL (5 μg/ml). Cells were fixed with 4% formaldehyde, stained with FITC labeled UEA-lectin and examined for uptake of DiI ac-LDL and FITC-lectin using an inverted fluorescence microscope under green and blue filters. Cells showing red fluorescence were considered to have taken up ac-LDL and lectin staining was observed with green fluorescence. An average of dual positive fluorescent cells were counted using object count feature of NIS-Elements (Software Version: 3.0), in 5–6 fields from duplicate wells of ALC/HC (*n* = 4 each) samples.

#### Cytokine profiling using multiplex bead-array assay

Supernatants of cultured EPCs from healthy controls and ALC patients were collected at day 8–10 of cell cultures. The cytokine concentrations in supernatants were determined by multiplex ELISA assay using MILLIPLEX MAP human cytokine magnetic bead panel (Millipore, Billerica, MA, USA) and manufacturer's protocol. The multiplex included pro-inflammatory and anti-inflammatory cytokines, and growth factors, such as IL-1β, IL-2, IL- 6, Il-4, IL-5, IL-15, IL-17A, IL-10, IFN-γ, TNF-α, VEGF, FGF, MIP-1b, RANTES, and GM-CSF. Standard curve was drawn using standards provided in the kit and each analyte concentration was calculated by logistic-5PL regression interpolation from the standard curve. The assays were normalized to 10^6^ EPCs per ml.

### Statistical analysis

Data were analyzed using the statistical software Prism (version 5; GraphPad Software) and are reported as mean ± standard deviation (*SD*). For analysis of the clinical data, Kruskal Wallis Test followed by probability adjustment by Mann–Whitney test was performed. Unpaired Student's *t*-test was performed to compare two mean values and *p* < 0.05 were considered statistically significant.

## Results

### Phenotype of endogenous circulating EPCs

#### Identification of EPCs by flow cytometry

**Figure 1:**
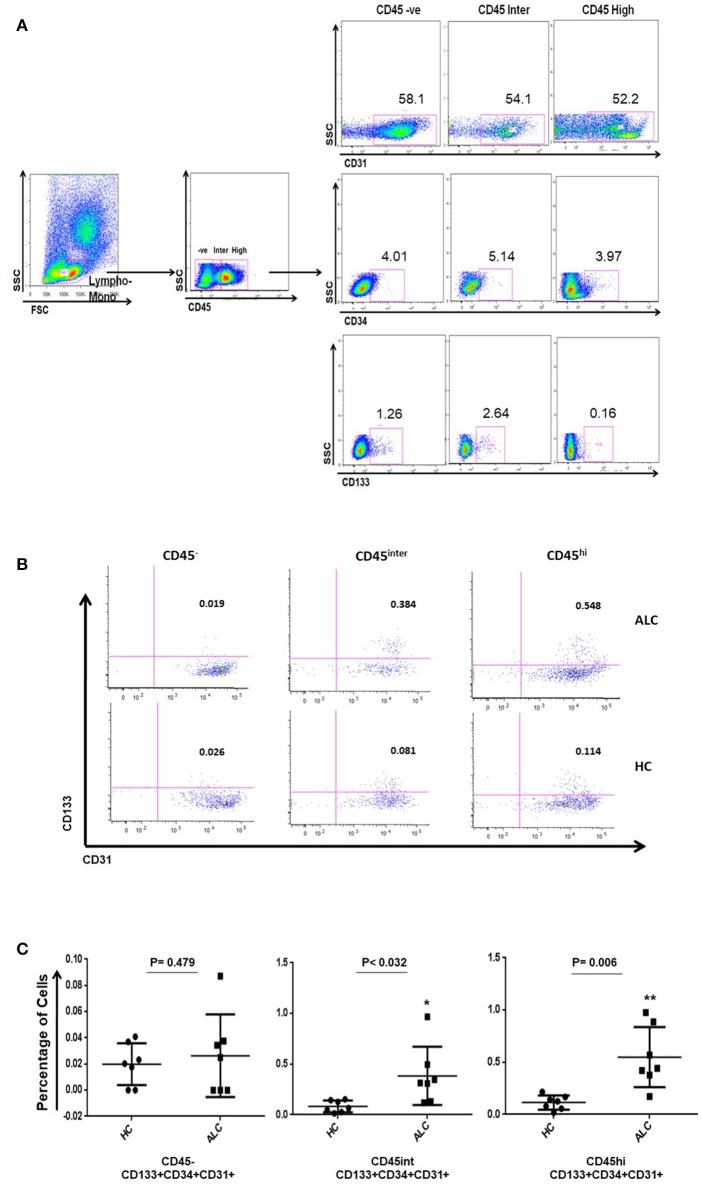
Identification of circulating EPC sub-populations in human samples by flow cytometry. **(A)** Gating strategy for identification of EPCs using CD34, CD133, CD31, and CD45 antibody markers. The CD45 population was gated on the overall lymphocyte+monocyte cells in the peripheral blood mononuclear cells (PBMC). Double positive cells (CD133+CD31+) were identified on CD34 positive gate in the three CD45 populations. **(B)** Based on CD45 expression (negative: CD45^−^; intermediate: CD45^int^; high: CD45^hi^), representative plots show three distinct sub-populations of EPCs (CD34+ CD133+ CD31+) in patients with alcoholic cirrhosis (ALC, upper panel) and healthy controls (HC, lower panel). **(C)** Dot plots show percentage of EPC sub-populations increase in CD45^int^ and CD45^hi^ EPCs in patients with alcoholic cirrhosis (*n* = 7) compared to healthy controls (*n* = 7). The percentages were calculated using backgate analysis and single color controls. ^*^P < 0.05; ^**^P < 0.01.

Circulating EPCs in the study subjects were identified as triple positives for CD34, CD133, and CD31 (Tanaka et al., 2017). Three distinct populations of circulating EPCs (CD34+ CD133+ CD31+) were observed based on their CD45 expression, namely CD45 negative (CD45^−^), CD45 intermediate (CD45^int^) and CD45 high (CD45^hi^) (Figures 1A,B). Within CD34+ populations expressing various levels of CD45, CD45^int^, and CD45^hi^ EPCs significantly increased in ALC patients compared to HC (*P*-val = 0.032, *P*-val = 0.006, respectively; Figure 1C).

#### Immunophenotyping of EPCs using mass cytometry

Analysis using the mass cytometer in independent cohort of patients and healthy controls also confirmed the CD45^−^, CD45^int^, and CD45^hi^ EPC sub-populations. Similar to flow cytometry results (Figure 1), maximum numbers of cells were obtained for CD45^int^ followed by CD45^hi^ EPCs with very few cells identified as CD45^−^ EPCs (Supplementary Figure 1B). Only a small percentage of cells expressed CD133 for both flow and CyTOF datasets. CD45 sub-populations showed minimal CD133 expression with CyTOF [Median ± *SD* (%): 5.1 ± 3.4, 7.8 ± 2.6, and 9.7 ± 8.7 for CD45^−^, CD45^int^, and CD45^hi^, respectively).

**Figure 2:**
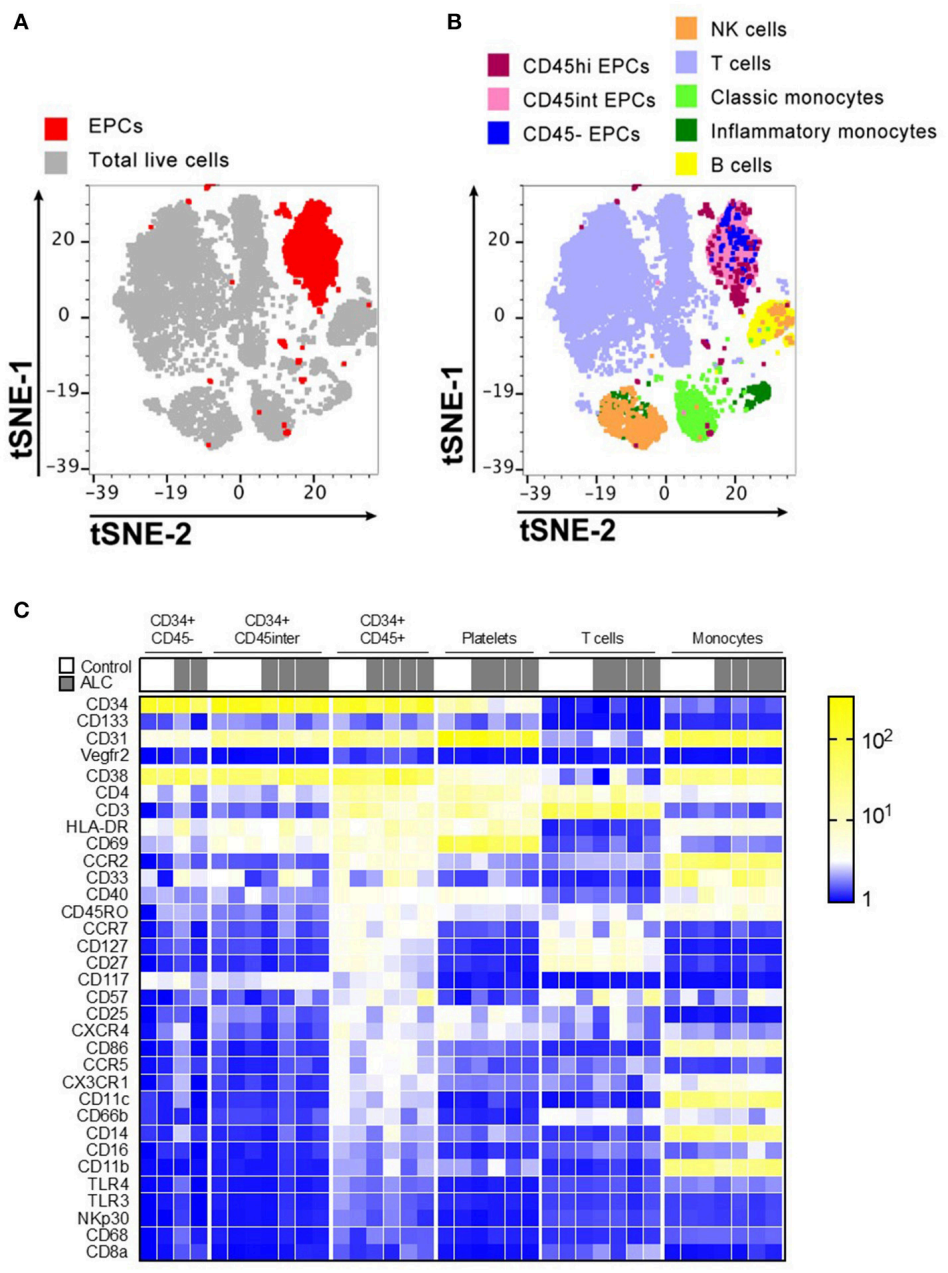
Immunophenotype characterization of EPC sub-populations by CyTOF. **(A)** t-stochastic neighborhood embedding (t-SNE) plot shows all EPC events (red; CD31+CD34+) occupy their own specific niche distinct from other immune populations. **(B)** Distribution of EPC sub-populations based on their CD45 expression, show that majority of EPCs were CD45hi and CD45^int^ with only ~5–6% of CD45^−^ EPCs. Plot also shows other immune cell populations in human PBMCs. **(C)** Mean Fluorescence Intensity Heatmap of full cohort showing variable expressions of immune markers on EPC sub-populations. T cells, platelets, monocytes for each sample is shown for comparison. Marker characteristic of EPC populations (CD34, CD133, CD31, and Vegfr2) shown at top, with remaining markers ordered by average intensity of marker expression for CD34+ CD45^hi^ population. Discrete expression patterns for the three EPC sub-populations show CD45^−^ and CD45^int^ EPCs sharing close resemblance that are markedly distinct from CD45^hi^ EPCs.

The visualization of CyTOF high-dimensional datasets using t-distributed Stochastic Neighbor Embedding (t-SNE) plots showed that CD31+CD34+ EPCs clustered in their own specific niche distinct from other immune populations (Figure 2A). Distribution of EPC sub-populations based on their CD45 expression showed that the majority of circulating EPCs largely occupied the same localization in high dimensional space (Figure 2B). Further, the t-SNE analysis showed that this localization of EPC subsets were independent from other major immune cell populations examined (T cells, B cells, NK cells, and monocytes).

Discrete patterns of expression (Mean Fluorescence Intensity Heatmap) were observed for the three EPC sub-populations, with CD45^−^ and CD45^int^ EPCs sharing close resemblance but markedly distinct from CD45^hi^ EPCs (Figure 2C). Unlike CD45^−^ and CD45^int^, CD45^hi^ EPCs predominantly expressed chemokine receptors (CCR2, CXCR4, CCR5, CCR7, CX3CR1) and several markers expressed by T cells (CD3, CD4, CD27, CD127, CD57, CD25, CD45RO) and myeloid cells (HLA-DR, CD11c, CD40, CD86). However, none of these three EPC sub-types expressed any Vegfr2. CD45^hi^ EPCs from ALC patients had decreased expression of CD127 and CD27 compared to HCs. No other major differences were noticed between controls and ALC patients for expression of the surface markers for any EPC sub-populations (Figure 2C).

Overall, major immune populations in PBMCs from ALC patients were distinct from controls. For example, B cells and CD4+ T cells significantly reduced in proportion of live peripheral mononuclear cells In ALC patients vs. HCs, whereas CD8+ T cells and monocytes increased, with significance evident only for CD8+ T cells (Supplementary Figure 2).

### *In vitro* functional characteristics of *ex vivo* EPC cultures

To functionally characterize the EPCs, PBMCs from controls and patients were cultured and expanded in *ex vivo* conditions using a cocktail of growth factors enriching EPCs.

**Figure 3:**
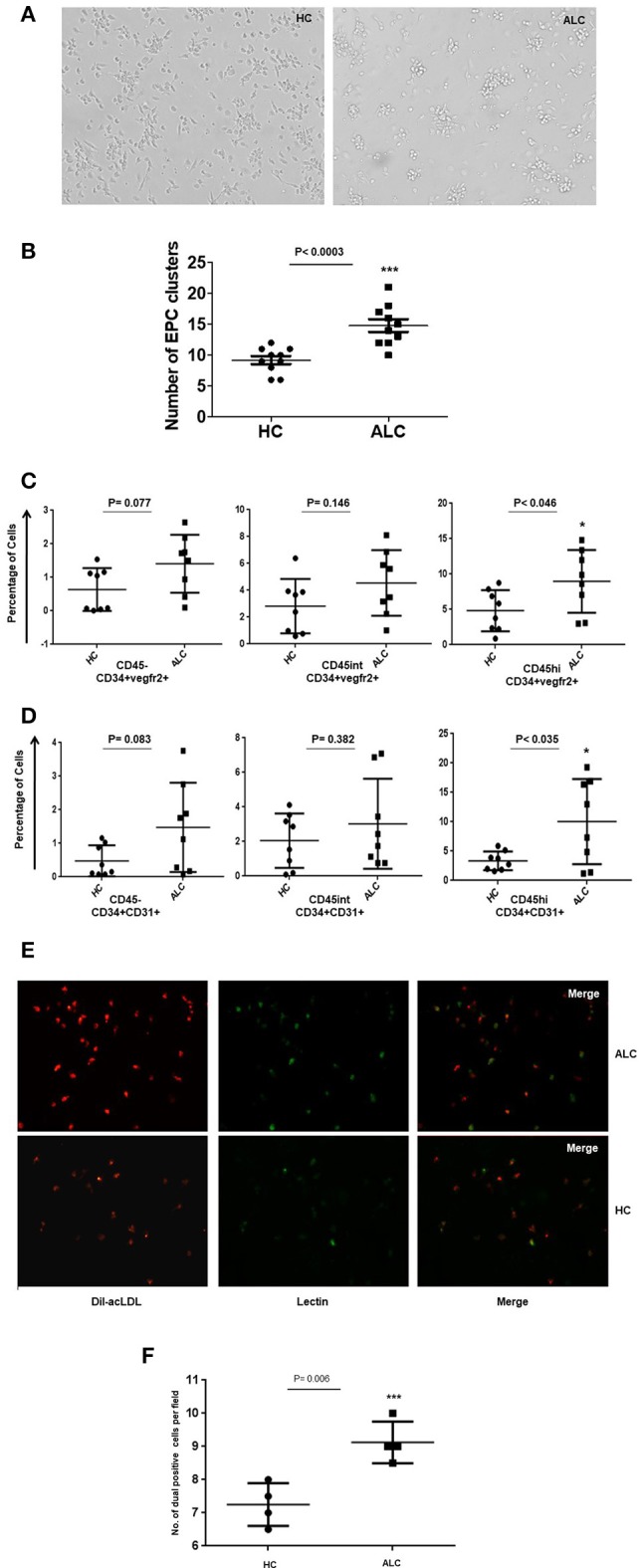
Morphological and functional characterization of *ex vivo* EPC in day 10 cultures. **(A)** Representative phase contrast images of EPC cultures at day 10 derived from PBMCs from healthy controls (HC) and patients (ALC). EPC clusters increased in ALC compared to HC (mag10X). **(B)** Dot plots of quantitation show significant increase in the number of EPC clusters in ALC patients compared to HC (*n* = 10 each). **(C)** Dot plots of cultured EPCs (CD34+vegfr2+) with CD45^−^/CD45^int^/CD45^hi^ sub-populations show significant increase of EPCs in ALC compared to HC only for CD45^hi^ EPCs. **(D)** Dot plots of cultured EPCs (CD34+CD31+) with CD45^−^/CD45^int^/CD45^hi^ sub-populations show significant increase of EPCs in ALC compared to HC only for CD45^hi^ EPCs. EPCs (CD34+vegfr2+; CD34+CD31+) were estimated as percentage of total cells (post-10 day cultures) by flow cytometry (*n* = 8 each). **(E)** Representative immunoflourescence images of cultured EPCs at day 10 show increased DiI-acLDL uptake (red) and UEA-lectin binding (green) and dual positives (yellow) in ALC patients compared to HC (mag 20X). **(F)** Significant increase in number of dual positive EPCs (ac-LDL and UEA-lectin) in patients with ALC compared to HC (*n* = 4 each). Dot plots show an average of about 5–6 fields from duplicate wells of ALC/HC samples counted for dual positive EPCs using object count feature of NIS-Elements (Software Version: 3.0). ^*^P < 0.05; ^***^P < 0.001.

Morphological characterization of EPCs at day 8–10 showed that the number of EPC colonies, defined as a cluster of round cells in the center and spindle-shaped cells at the periphery (Hill et al., 2003), significantly increased in EPCs from ALC patients compared to those from healthy controls (*p*-val = 0.003, Figures 3A,B). EPCs in culture were defined as CD34+CD31+ or CD34+Vegfr2+ cells. We did not phenotype these cells for CD133 as its expression is lost in cultured cells. Similar to the circulating EPCs, three sub-populations (CD45, CD45^int^, and CD45^hi^) were observed for the cultured EPCs at day 10. Results revealed that the percentage of CD45^hi^ EPCs (CD34+Vegfr2+ and CD34+CD31+) from ALC patients significantly increased (*P*-val 0.046 and *P*-val 0.035, respectively) compared to those from controls. No difference between HC and ALC groups was observed for CD45^−^ and CD45^int^EPCs (Figures 3C,D).

The ac-LDL uptake and lectin binding, measured to test the functionality of cultured EPCs, showed substantially higher number of day 10 EPCs with ac-LDL uptake from ALC patients compared to those derived from healthy controls (Figure 3E). Furthermore, a significant (*P*-val 0.006) proportion of ac-LDL positive cells co-expressed lectin-binding in EPCs from ALC patients compared to those from controls (Figure 3F).

### Secretory phenotype of cultured EPCs in patients with ALC by ELISA

**Figure 4:**
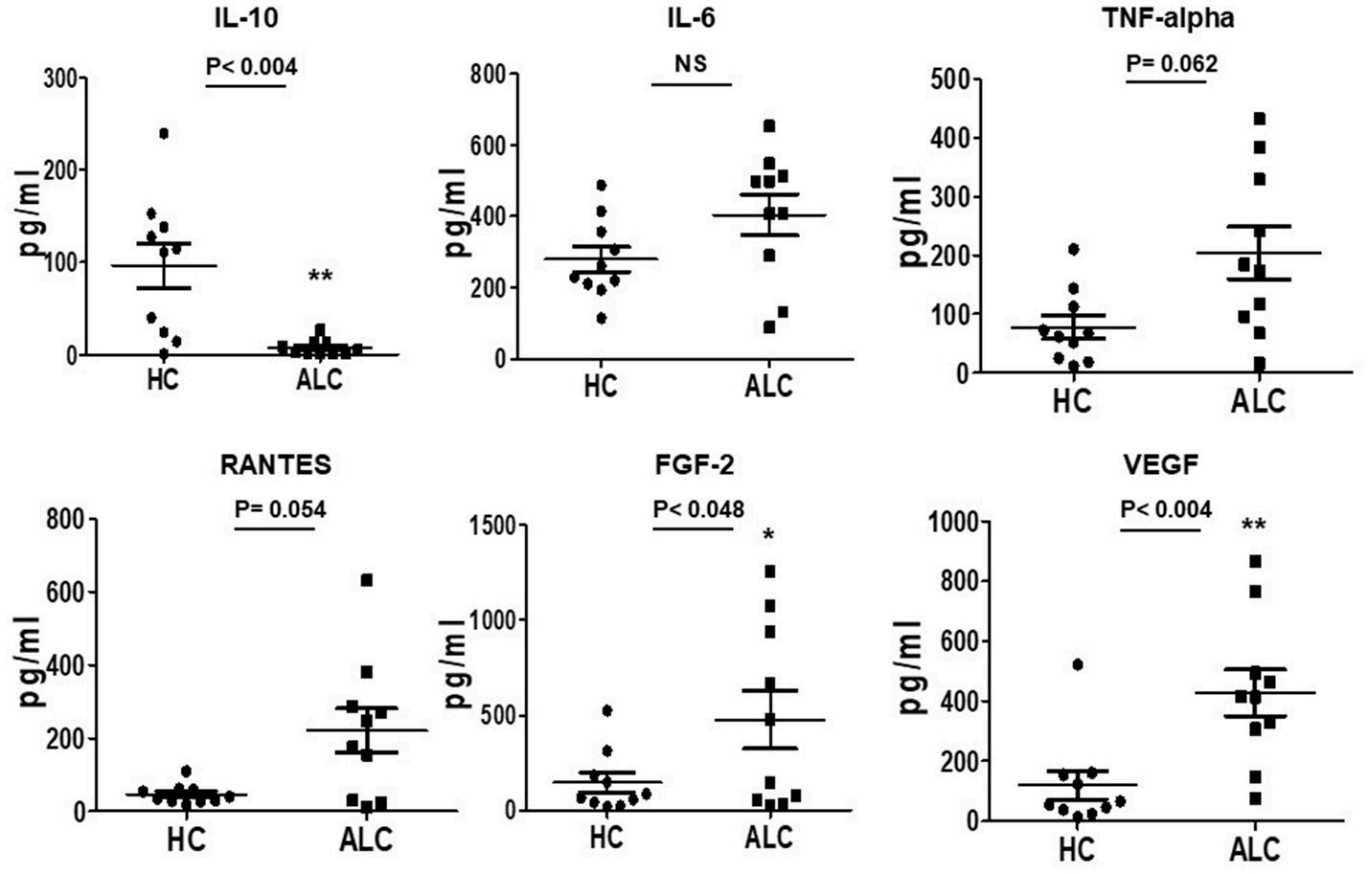
Secreted cytokine profiles of cultured EPCs from ALC patients and HC at day 10. Dot plots of normalized levels of cytokines, chemokines, and growth factors (pg/ml) show significant up-regulation in the secretion of FGF-2 and VEGF, and down-regulation of IL-10 in patients with ALC (*n* = 10) compared to healthy controls (*n* = 10). ^*^P < 0.05; ^**^P < 0.02. NS, Non-significant.

Investigations on secretory functions of cultured EPCs showed significant downregulation of anti-inflammatory cytokine IL-10 (*P*-val 0.004). Up-regulation in the secretion of pro-inflammatory cytokines, TNF-alpha and chemokine, RANTES (CCL5) was evident but did not reach significance. Furthermore, angiogenic growth factors FGF-2 and VEGF were significantly (*P*-val 0.048 and 0.004, respectively) upregulated in supernatants from patient EPCs compared to EPCs from healthy controls (Figure 4).

## Discussion

This study reports the first extensive characterization of the rare circulating human EPCs in patients with ALC and age-matched healthy controls. EPCs, defined as CD34+CD133+CD31+ had three phenotypically distinct subsets based on the CD45 expression, namely CD45^hi^, CD45^int^, and CD45^−^. Interestingly, CD45^hi^ EPCs that were significantly increased in patients with ALC compared to controls, also expressed T cell and myeloid markers, and chemokine receptors indicative of an increased activation of hematopoietic lineage of angiogenic cells in ALC. Furthermore, *ex vivo* EPC cultures from ALC patients also had significantly greater levels of CD45^hi^ cells coinciding with markers for EPC activation, such as increased uptake of ac-LDL, lectin binding and secretion of angiogenic mediators (FGF-2, VEGF). In addition, significantly reduced secretion of anti-inflammatory IL-10 and a trend for increased proinflammatory cytokines (TNF-alpha, RANTES) in EPCs from ALC patients, suggests specific inflammatory functions of these cells in this disease, supporting the proinflammatory phenotype of EPCs reported in other pathologies (Loomans et al., 2009; Zhang et al., 2009).

EPCs were first identified as circulating angiogenic cells by the co-expression of hematopoietic and endothelial markers, CD34 and Vegfr2 in 1997 (Asahara et al., 1997), but due to their heterogeneous nature and the disparate results in different clinical studies, the true identity of these cells remains elusive. Our study using ~36 metal tagged antibodies simultaneously (CyTOF), provides for the first time a detailed phenotype of circulating EPCs in clinical samples. Overall, the EPC sub-populations from ALC patients and healthy controls had similar phenotypic profiles. We used t-SNE, a powerful visualization tool for high dimensional datasets, and showed that circulating EPCs clearly clustered in their own niche distinct from other immune cells, and were enriched for CD45+ population, with CD45^hi^ sup-population in particular showing a heterogeneous distribution across other immune cell markers.

The presence of CD45 on EPCs has been controversial. CD45^−^ population is usually defined as true EPCs with endothelial function and CD45^+^ EPCs to belong to hematopoietic lineage (Timmermans et al., 2009; Yoder, 2012). We analyzed both, CD45^−^ and CD45+ (CD45^int^ and CD45^hi^) EPCs to specifically delineate the differences between these populations. We observed that CD45^int^ CD34+CD133+CD31+ and CD45^hi^ CD34+CD133+CD31+ EPCs increased in ALC patients, indicating that this chronic and severe disease state may specifically be triggering the migration of CD45^+^ hematopoietic EPC sub-population in circulation.

Distinct from CD45^−^ and CD45^int^ cells, the circulating CD45^hi^ EPCs also expressed chemokine/cytokine, T cell, and myeloid cell receptors (CCR2, CCR5, CXCR4, CX3CR1, CD3, CD4, CD69, CD11c), further indicating discrete functions (migration, trafficking, immune response activation) of the CD45^hi^ fraction of EPCs. Similar to our observations, others reported cultured BM-derived EPCs expressing CD45 had high proliferative potential, expressed myeloid markers and growth factors (Wang et al., 2011). Our results also corroborate earlier reports where CCR5 and CX3CR1 were shown to be expressed on activated EPCs and involved in EPC homing and migration from the circulation to injured sites (Walter et al., 2005; Ishida et al., 2012). In particular, expression of CX3CR1, a specific receptor for the novel chemokine fractalkine (FKN, also known as CX3CL1) (Herlea-Pana et al., 2015), on CD45^hi^ is intriguing, for this receptor-ligand binding has a role in leukocyte trafficking, vessel patrolling and as a potential target for inflammatory conditions. Our data with increased migration of CD45^+^ EPCs in circulation may represent a compensatory mechanism to support the reparative and regenerative capacity of the cirrhotic liver and needs further testing. We also discovered that CD45^hi^ EPCs had decreased expression of CD27 (TNF-alpha superfamily member regulating T-cell and B-cell activation) and CD127 (IL-7 receptor) in ALC patients compared to the healthy subjects. The precise role of CD27 and CD127 on EPCs warrants further investigation in the pathogenesis of ALC.

Regardless of CD45 expression, all three sub-populations of circulating EPCs had high CD31 and low CD133 expression, observed both by flow and CyTOF experiments. The *ex vivo* cultured EPCs had significant Vegfr2 expression but no detectable CD133, suggesting an endothelial commitment for these cells. These results are concordant with the recent study reporting expression of CD133 and Vegfr2 to be mutually exclusive (Case et al., 2007; Lanuti et al., 2016).

Previous studies reported the presence of two populations of EPCs in culture, early EPCs (2–3 weeks) and late outgrowth EPCs (OECs, 4–8 weeks; Hur et al., 2004). The early EPCs with high CD45 expression represent hematopoietic cells and a monocytic-like molecular profile, while the OECs with low CD45 expression with a molecular fingerprint suggestive of endothelial lineage (Hur et al., 2004; Ingram et al., 2004; Yoder et al., 2007; Medina et al., 2010). Our *ex vivo* cell culture studies validated that patients with ALC retained EPCs with high CD45 positivity as well as increased colony formation in comparison to the controls. Since our day 10 cultured cells can be considered early EPCs, a high percentage of CD45 positive cells were expected, but the significant increase in CD45^hi^ EPCs in ALC patients is a novel and unexpected discovery.

Furthermore, cultured EPCs from ALC patients secreted high levels of pro-inflammatory cytokines including TNF-alpha and RANTES, and angiogenic growth factors, including FGF-2 and VEGF, compared to control EPCs. These enhanced levels of inflammatory cytokines and growth factors are likely due to the increased proportion of CD45+ (CD45^hi^ and CD45^int^) EPCs in ALC patients. It may thus be hypothesized that in ALC, there exists an increased population of inflammatory CD45+ EPCs in circulation, which may not only participate in angiogenic repair but also act as pro-inflammatory mediators (Zhang et al., 2009). Further, circulating EPC mediated angiogenesis *per se* may not only initiate tissue repair in the liver but also contribute to inflammation and damage (Kaur and Anita, 2013). Whether this is also true for liver disease etiologies other than alcohol warrants further investigations.

In summary, we report the first comprehensive characterization and identification of distinct categories of circulating human EPCs. Our study suggests that CD45^hi^ EPCs are angiogenic cells expressing inflammatory chemokines and immune cell receptors that are distinct from the CD45^−^ subset. Further, significant increase in circulating CD45^hi^ EPCs in ALC patients may contribute to alcoholic liver injury via paracrine secretion of inflammatory and angiogenic mediators and represent a systemic pro-inflammatory state affecting the progression of the disease.

## Ethics statement

The study was approved by the institutional ethics committee of the Institute of Liver and Biliary Sciences (ILBS), New Delhi, and informed written consent was obtained from all the subjects. For the Australian recruitment, informed written consent was obtained from all subjects under protocol HREC/11/RPAH/88 and HREC/15/RPAH/380 approved by Sydney Local Health District Human Research Ethics Committee. Informed consent was in accordance with the Declaration of Helsinki. No minors or vulnerable populations were involved.

## Author contributions

NT and DS: designed, collected specimens, and led the overall study; SK and DS: led the writing of the manuscript; RS and SK: conducted laboratory experiments; SMS: provided statistical expertise; HM: performed CyTOF in consultation with DS; Intellectual input for reviewing and presenting data was provided by DS (methodologies), BF (CyTOF), SMS, GM, and SS (clinical).

### Conflict of interest statement

The authors declare that the research was conducted in the absence of any commercial or financial relationships that could be construed as a potential conflict of interest.

## Supplementary material

Supplementary Figure S1 **(A)** Gating of the PBMCs in the lymphocyte and monocyte region with single color SSC plots for different flourochromes used as controls in the study. **(B)** Gating strategy for CyTOF using CD34, CD31 and CD45 markers. (a) platelets, (b) CD34+CD45- population, (c) CD34+CD45^int^ population and (d) CD34+CD45^hi^ population.

Supplementary Figure S2 Major immune populations in PBMCs from ALC patients and controls (HC). Compared to HC, B cells and CD4+ T cells were significantly downregulated, CD8+ T cells and monocytes were upregulated in ALC, reaching significance only for CD8+ T cells. ^*^*p*-val = < 0.05; ^**^*p*-val = < 0.02.
