# Supplementary figures and images for: Circulating Endothelial Progenitor Cells Present an Inflammatory Phenotype and Function in Patients With Alcoholic Liver Cirrhosis
Source: Front Physiol. 2018 May 22;9:556. doi: 10.3389/fphys.2018.00556 (PMC5972283; doi:10.3389/fphys.2018.00556)

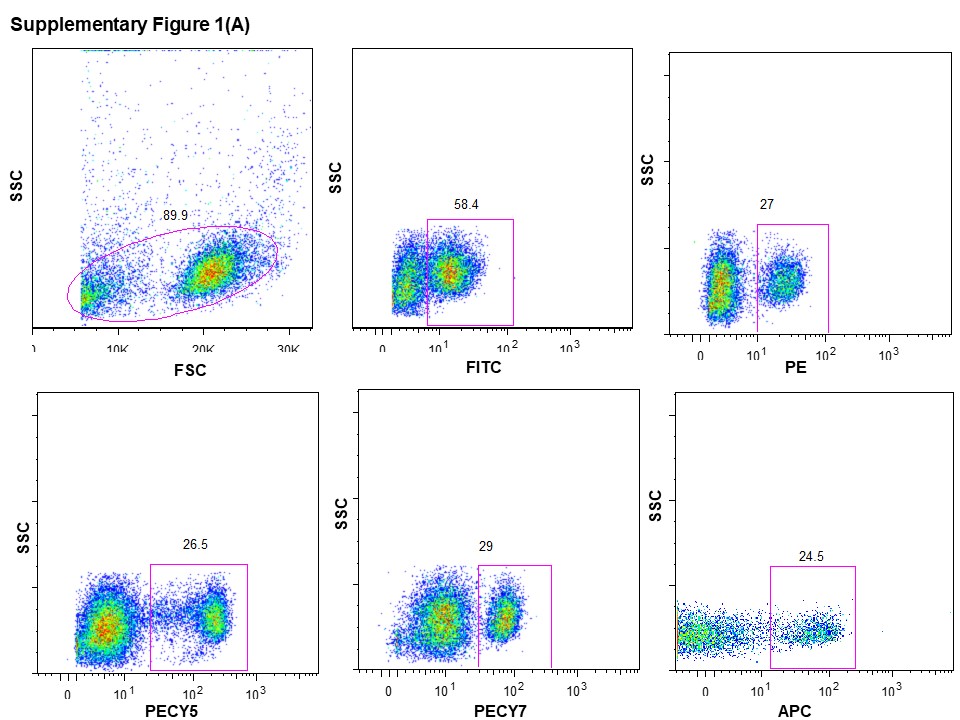

Supplement: Supplementary Figure S1 — (A) Gating of the PBMCs in the lymphocyte and monocyte region with single color SSC plots for different flourochromes used as controls in the study. (B) Gating strategy for CyTOF using CD34, CD31 and CD45 markers. (a) platelets, (b) CD34+CD45- population, (c) CD34+CD45int population and (d) CD34+CD45hi population. [file Image_1.JPEG]

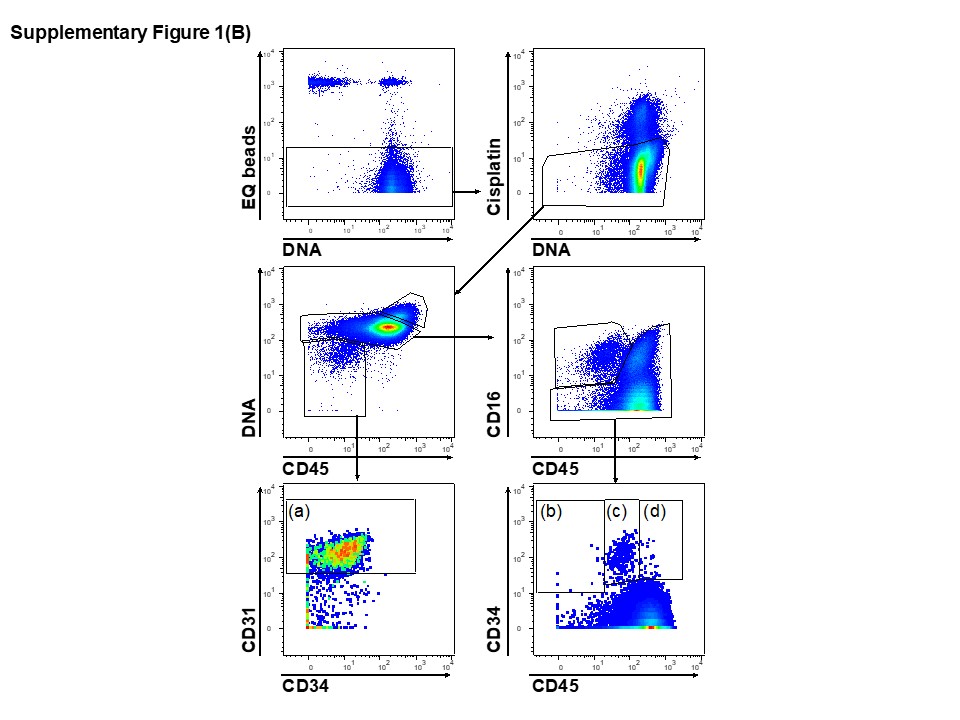

Supplement: Supplementary file 2 [file Image_2.JPEG]

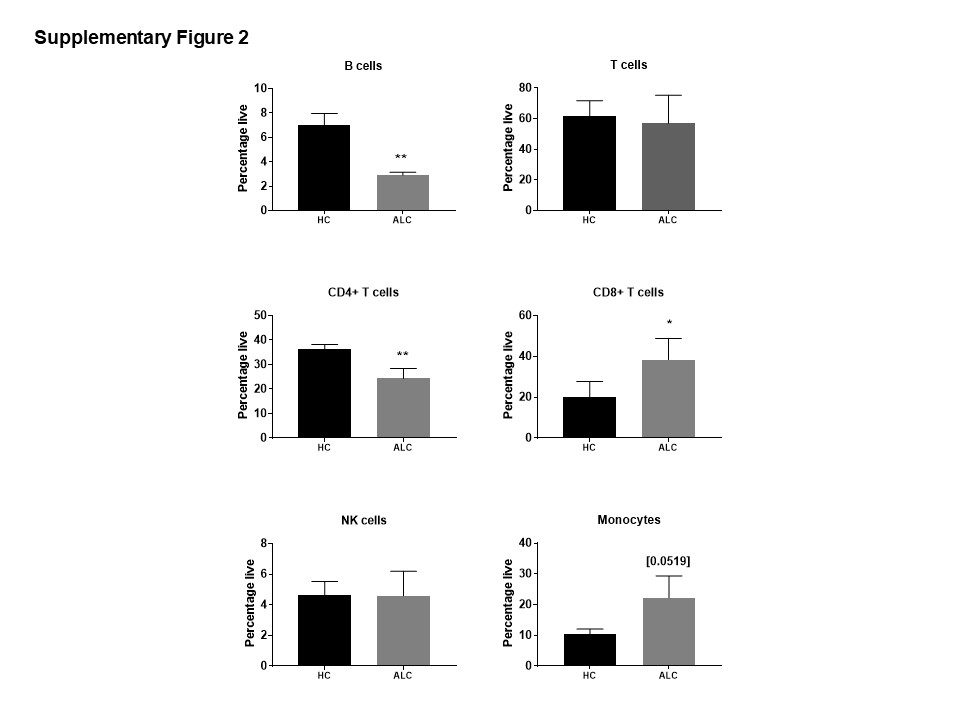

Supplement: Supplementary Figure S2 — Major immune populations in PBMCs from ALC patients and controls (HC). Compared to HC, B cells and CD4+ T cells were significantly downregulated, CD8+ T cells and monocytes were upregulated in ALC, reaching significance only for CD8+ T cells. *p-val = < 0.05; **p-val = < 0.02. [file Image_3.JPEG]
